# Physiological impact of load carriage exercise: Current understanding and future research directions

**DOI:** 10.14814/phy2.15502

**Published:** 2022-11-02

**Authors:** Mark A. Faghy, Ren‐Jay Shei, Nicola C. D. Armstrong, Mark White, Mitch Lomax

**Affiliations:** ^1^ Biomedical Research Theme, School of Human Sciences University of Derby Derby UK; ^2^ Division of Pulmonary, Allergy, and Critical Care Medicine, Department of Medicine University of Alabama at Birmingham Birmingham Alabama USA; ^3^ Defence Science and Technology Laboratory Salisbury UK; ^4^ Extreme Environments Laboratory, School of Sport, Health and Exercise Science University of Portsmouth Portsmouth UK; ^5^ Rocky Mountain University of Health Professions Provo Utah USA

**Keywords:** backpack, ergonomics, exercise tolerance, load carriage, occupational physiology, military physiology, respiratory muscles, weighted exercise, personal protective equipment

## Abstract

Load carriage (LC) refers to the use of personal protective equipment (PPE) and/or load‐bearing apparatus that is mostly worn over the thoracic cavity. A commonplace task across various physically demanding occupational groups, the mass being carried during LC duties can approach the wearer's body mass. When compared to unloaded exercise, LC imposes additional physiological stress that negatively impacts the respiratory system by restricting chest wall movement and altering ventilatory mechanics as well as circulatory responses. Consequently, LC activities accelerate the development of fatigue in the respiratory muscles and reduce exercise performance in occupational tasks. Therefore, understanding the implications of LC and the effects specific factors have on physiological capacities during LC activity are important to the implementation of effective mitigation strategies to ameliorate the detrimental effects of thoracic LC. Accordingly, this review highlights the current physiological understanding of LC activities and outlines the knowledge and efficacy of current interventions and research that have attempted to improve LC performance, whilst also highlighting pertinent knowledge gaps that must be explored via future research activities.

## Graphical abstract

This report provides a synthesis of the historic and recent research in occupational load carriage to bring together the current understanding and future direction for research in this important area.

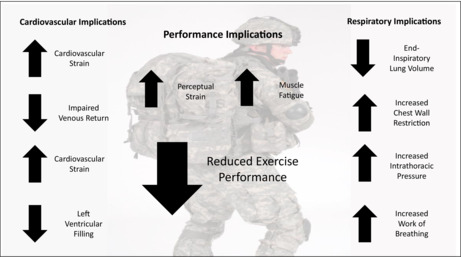

## 1 INTRODUCTION

Load carriage is an essential part of many physically demanding occupations, with the heaviest loads carried by first responders and military personnel; indeed there are examples where loads carried by infanteers have approached their body mass (Armstrong et al., 2019; Lloyd‐Williams & Fordy, 2013). In occupational groups, a load can be carried on the head (helmet), hips (webbing belt, specialist equipment), arms/legs (extremity armor), hands (weapons and hoses) and feet (boots), but the largest proportion of the load is carried on the torso (backpack, body armor, webbing pouches, breathing apparatus). It is well established that LC has a detrimental effect on task performance yet the loads carried by occupational groups continue to rise (Knapik et al., 2004). These performance decrements are influenced by the characteristics of the load carried including mass, coverage, rigidity, bulk and fit (Choi, Garlie, et al., 2016). The impact of these characteristics on the wearer's physiology, biomechanics, cognition and health has been the subject of investigation in an attempt to reduce injury risk, improve the design of LC equipment as well as optimize the performance of load carriers (Liew et al., 2016; Orr et al., 2014; Taylor et al., 2016; Walsh & Low, 2021). As the characteristics of LC systems, personnel and occupational tasks continue to evolve, consolidation of current understanding is needed to inform the design and development of ergonomic research and systems that can improve PPE and LC systems that optimize performance and reduce it imposes on the wearer.

The very nature of LC activities imposes elastic and inertial forces upon the thoracic cavity leading to shoulder tissue deformation (Hadid et al., 2015) and restricted movement of the chest wall and ribcage which, increases total physical work and compromises exercise capacity. Recent investigations exploring the specific physiological impairments caused by LC have focused on the ventilatory and cardiopulmonary effects of LC at rest and during exercise. These studies have demonstrated alterations in breathing mechanics (Phillips, Stickland, Lesser, et al., 2016) and quantified respiratory muscle fatigue (RMF) with LC through measurement of mouth pressures before and after load carriage (Faghy et al., 2016; Hinde et al., 2018; Phillips, Stickland, Lesser, et al., 2016). LC presents a unique challenge to the respiratory system which may account for the decrements in task performance observed during LC performance.

This review examines existing research describing the physiological consequences of LC activities and provides a critical appraisal of the different methodological approaches used to date. This review will consider the physiological mechanisms by which torso‐borne LC negatively impacts performance and identify knowledge gaps to inform the direction and methodology of future investigation in this area. Further, this review will update knowledge relating to strategies that may minimize the effects of LC on the physical capacity and performance of occupational load carriers.

Studies were identified by searching the following databases: PubMed, Google Scholar, and Web of Science for English‐language articles investigating the effects of LC on exercise performance, and physiological measures. Search terms included, but were not limited to, ‘load carriage’, ‘backpack’, ‘weighted vest’, ‘chest‐wall restriction’, ‘exercise capacity’, ‘performance’, ‘gait’, ‘respiratory muscles’, ‘fatigue’, ‘sex’ and ‘training’. After the initial identification of relevant articles, the reference lists of all selected articles were searched for additional relevant papers.

## 2 CARDIOVASCULAR EFFECTS OF LOAD CARRIAGE

Load carriage alters the cardiovascular response to exercise both directly and indirectly. Directly, the physical confines of LC induce mechanical compression of the thorax. Indirectly, the added mass of LC increases the metabolic requirements of the task. These factors may be further affected by the characteristics of the LC (bulk, coverage, distribution), the task being undertaken (incline, terrain, speed, duration), the environment (climate, altitude) and individual factors such as fitness level and descriptive characteristic (stature, body composition, sex).

## 3 DIRECT EFFECTS

The largest volume of LC is typically carried around the shoulder girdle (i.e., shoulders) and thoracic cavity (torso). Therefore, the direct mechanical pressure of LC causes shoulder tissue deformation and chest wall restriction (CWR), reducing sensory (i.e., brachial plexus) and blood flow (subclavian artery supply) to the arms (Hadid et al., 2015), while simultaneously increasing intrathoracic pressure (Coast et al., 1990). Shoulder tissue deformation has been demonstrated with loads as light as 12 kg and durations as short as 10 min, with the resultant effects being reduced blood supply and sensory impairment (light‐touch) to the upper limb digits and fine‐motor skills (Hadid et al., 2017; Kim et al., 2014). The implication of increased forces being placed upon the shoulders due to strap forces can result in physiological implications such as altered biomechanics with increased energy cost resulting in upper body muscle fatigue, higher rates of breathing discomfort (Phillips, Stickland, Lesser, et al., 2016), and shoulder palsy which have downstream implications to occupational duties primarily dependent upon the shoulder girdle; for instance, marksmanship and throwing of grenades (Choi, Blake Mitchell, et al., 2016; Knapik et al., 2004). To mitigate these negative effects, it is recommended that the maximum forces applied to each shoulder should not exceed 145 N and maximum pressures 20 kPa (2.0 N cm^−2^) (Stevenson et al., 2004).

Separately, CWR can reduce the magnitude of negative pressure swings in the thorax (Miller et al., 2005), which reduces the size of the pressure gradient for blood returning to the heart and impairs venous return and exercise performance (Phillips, Stickland, Lesser, et al., 2016). Cardiac preload can also be reduced by external compression of the chest wall, coupled with inflation of the lung, which can compress the vena cava (Nelson et al., 2009). These factors can be further exacerbated by the forward lean posture often adopted during LC exercise (Seay, 2015), as well as LC‐related increases in circulating catecholamines that raise systolic blood pressure (SBP), increase afterload, and increase rate pressure product (workload of the heart; Miller et al., 2005), factors which may have a deleterious effect on left ventricular function. Previously, Miller et al. (2002) observed decreased left ventricular filling and emptying rates with CWR, leading to a 16%–20% decrease in stroke volume and a 12% decrease in cardiac output during submaximal cycling. Nelson et al. (2009) observed similar effects with a self‐contained breathing apparatus; however, this finding was shown to be a resultant effect under specific compounding conditions; that is, exercise time, plus heat stress and dehydration conditions; meaning this effect may not be applicable across all LC circumstances. Sagiv et al. (2006) found no difference in left ventricular systolic function during loaded treadmill walking in adolescent, adult, and elderly participants, respectively. Regarding arterial stiffness, Ribeiro et al. (2014) measured the augmentation index (AIx), which is an independent predictor of cardiovascular events and mortality. In healthy young participants just 10 min with carrying loads of 10% of body mass, caused significant increases in AIx.

## 4 INDIRECT EFFECTS OF LOAD, SPEED, AND BODY COMPOSITION

The additional mass, bulk and coverage of LC increases metabolic demand for the same exercise task, which in turn increases cardiovascular strain. Increases in the rate of oxygen uptake (*V̇*O_2_), accompanied by increases in heart rate (HR), have been widely shown during submaximal LC (Armstrong et al., 2019; Dominelli & Sheel, 2012; Sol et al., 2018). These increases occur for aerobic exercise lasting from 10 min (Majumdar et al., 1997) to 4 h (Larsen et al., 2011). Studies that do not show increases in *V̇*O_2_ with LC use self‐paced exercise protocols. During self‐paced tasks participants typically reduce their speed to cope with the additional demands of LC leading to a reduction in *V̇*O_2_. However, overall work done is greater as participants need to work for longer to complete the task (Simpson et al., 2011) which coupled with a gradual increase in *V̇*O_2_ over time (Patton et al., 1991) leads to an increase in overall work done.

Indices of body composition as well as absolute aerobic power influence the relative metabolic demands of LC (Lyons et al., 2005). The reasons for these increases are two‐fold: (1) the increased postural demand of LC requires greater isometric activation of torso muscles (Shei et al., 2017), and (2) the added mass requires more work by the locomotor muscles to maintain the same speed (Boffey et al., 2019). Because the overall workload at any given speed will be greater in LC, relative speeds determined by unloaded maximal tests (50% of speed at unloaded *V̇*O_2peak_) may underestimate exercise intensity during LC exercise. *V̇*O_2peak_ values may be similar between loaded and unloaded conditions, but if they occur at similar workloads this will equate to a lower maximal speed in LC (Swearingen et al., 2018). This mismatch of exercise intensity has been demonstrated by an increased rate of perceived exertion (RPE) and local muscle fatigue during LC compared to unloaded, at the same absolute speed (Faghy & Brown, 2014a), and separately by the decreased absolute speed at matched *V̇*O_2_ levels (Shei et al., 2018). Phillips, Stickland, Lesser, et al. (2016) found RPE and leg fatigue were greater with a 25 kg backpack versus no‐load despite matched oxygen demand (~3 L min^−1^) and attributed this to the increased mass carried leading to increased muscle recruitment and a change in posture. The authors also noted that breathing discomfort was greater during LC in the last 20 min (although discomfort vs. minute ventilatory pattern was unchanged).

Current literature also recommends that to delay time to fatigue during sub‐maximal exercise (prolonged marching activity), load and speed should be carefully managed to maintain an exercise intensity ~45% *V̇*O_2_ max (Johnson et al., 2021). Furthermore, a study deriving the load–speed index (LSI), corroborated this finding, identifying 47% *V̇*O_2_ max as a threshold above which intensity increases at a greater rate with increases in two critical factors of load carriage; i.e., load and speed (Boffey et al., 2019). Of course, the applicability and utilization of LSI should be considered carefully before implementation; for example, within a military education and training pipeline, LSI might be a useful tool to teach the relationship between an individual's physical ability (aerobic capacity), their biomechanical efficiency relative to the mass of the load (trunk lean, gait variables), and the amount of energy required for the task of the associated characteristics of LC. However, LSI will have limited operational feasibility within a Forward Operating Area, given that the LC and speed will vary dependent on the mission and associated operational tasks.

It is well established that environmental stressors (heat, cold and altitude) alone increase cardiovascular strain (Åstrand et al., 2003). Thus, when combined with LC these stressors will further exacerbate the increased cardiovascular strain experienced with LC. At increased ambient temperatures and/or humidity's cardiovascular strain under LC is increased when compared to unloaded conditions due to the increased energy demand of LC which leads to increased metabolic heat production. Further, the increased coverage of LC will reduce heat dissipation. This impaired thermoregulation during LC will increase sweat rate (without an increase in sweat evaporation) and loss of plasma volume (Nelson et al., 2009), ultimately leading to greater cardiovascular strain (Caldwell et al., 2011; Majumdar et al., 1997).

Increased energy demands with LC in cold environments have also been reported; at −10°C a 20% increase in $\dot{V}$O_2_ was observed when compared to measurements taken at 20°C (Hinde et al., 2018). Operating at altitude combines the stress of cold environments with reduced barometric pressures which further adds to the cardiovascular demands of LC. Chatterjee and colleagues (2017) reported an increase in $\dot{V}$O_2_ of up to 14% at higher altitudes when acclimated participants marched with 30 kg at 3500 and 4300 m (Chatterjee et al., 2018).

## 5 THE INFLUENCE OF MASS, DISTRIBUTION, AND TERRAIN

The direct mechanical and indirect metabolic effects of LC may be further influenced by factors such as the mass of the load, the distribution of the load, and the speed and grade of exercise. The effect of absolute mass may be non‐linear, as Lyons et al. (2005) found a 20–40 kg (25%–50% body mass) change in load to elicit a greater increase in HR and *V̇*O_2_ compared to a 0–20 kg change (0%–25% body mass). Conversely, Beekley et al. (2007) found increases in *V̇*O_2_ and HR to be linear with increases in relative load from 0% to 70% lean body mass. It should be noted that most LC studies report changes in mass carried, but the bulk and coverage of the LC are not characterized, often because of a lack of available methods to do this. As such, it is challenging to quantify the impact of load mass alone given that increases in mass carried will also be accompanied by an increase in bulk and coverage.

While it is evident that the mass of the LC plays a significant role in the response of the cardiovascular system (CVS), the distribution of the LC on the torso is also important in determining the overall level of CWR, postural stability, and the increase in metabolic demand of LC exerTorso‐borne borne load carried by military personnel is not limited to backpacks, it also includes webbing and body armor. Early work has shown that load carried as a double pack distributed across the chest and back reduces the metabolic cost of load carriage by 9% compared to a traditional backpack (Datta & Ramanathan, 1971). Whilst carrying a load close to the centre of mass may reduce metabolic cost, the trade‐off is greater for CWR as this design reduced maximal voluntary ventilation (MVV) by 10% more than backpack loads (Legg & Mahanty, 1985). The position of the load in a backpack is also an important consideration. Stuempfle et al. (2004) found load carried on the lower back (lumbar vertebrae 1–5) to elicit greater increases in *V̇*O_2_ compared to the upper back (thoracic vertebrae 1–5; 18.6 ± 2.3 vs. 22.2 ± 3.0 ml kg^−1^ min^−1^ respectively) a finding which has been confirmed by others (Abe et al., 2008).

In addition to the mass and the placement of the load, the slope of the terrain can affect the cardiovascular response. Indeed, Sagiv et al. (2000) concluded gradient has a larger effect than load mass in determining the cardiovascular response. Uphill exercise elicits a greater metabolic demand and thus *V̇*O_2_ compared to level exercise (5.11 ± 0.89 vs. 7.36 ± 0.95 Kcal min^−1^), and this effect is linear with grade (Chatterjee et al., 2018). However, LC may not affect this relationship until slopes 6% or greater, when LC elicits an increased *V̇*O_2_ over non‐LC exercise at or above this incline (Phillips, Stickland, Lesser, et al., 2016). Downhill LC elicits a lower overall metabolic demand, but when expressed as a percent increase from baseline, downhill LC exercise may result in greater increases in *V̇*O_2_ across the exercise task compared to level exercise (Blacker et al., 2009; Chatterjee et al., 2018). This could be due to the greater *V̇*O_2_ drift associated with eccentric exercise. Also, the speed of movement under LC has a massive impact on CVS measures. In elite soldiers carrying a 20 kg backpack an increase from 6.4 to 7.4 km h^−1^ raised mean HR by 20 bpm^−1^ (Simpson et al., 2011). Paul et al. (2015) found an almost linear increase in HR and $\dot{V}$O_2_ with gradient increases (0%–25%) and walking speed (2.5–4.0 km h^−1^) with increasing loads of 16.1%–32.2% of body mass.

From the research presented it seems evident that LC significantly increases the demands of the heart versus no LC, which may alter left ventricular systolic function. However, Sagiv et al. (1993) found that carrying 53% and 66% of one's body mass for 4 h demonstrated a steady‐state in left ventricular function (similar ejection fraction and stroke volume) but different HR and blood pressure (BP) responses between groups as rate pressure product was significantly elevated in the 66% body mass group. At the end of 240 min of exercise, mean arterial BP were 92 mmHg in the 53% body mass condition and 99 mmHg in the 66% body mass condition. It should be noted that the treadmill walking was with no gradient and at a constant speed (Sagiv et al., 1993). Sagiv et al. (2000) also concluded that during movement with LC, the prime determinant of CVS demand is changes in gradient compared to LC mass. Further, Drain et al. (2016) reported that when all factors are held constant, walking speed is a more robust mediator of work output than LC mass. These findings of steady left ventricular systolic function were replicated by Sagiv (2001) and Sagiv et al. (2006) in both elderly and adolescent participants. In these studies, participants did not show differences in left ventricular function with heavier LC versus no mass as ejection fraction, SV, end‐diastolic and systolic volumes stayed similar, although BP and HR (along with $\dot{V}$O_2_ in the adolescents) increased with the LC in comparison to no or lighter LC mass.

Regarding the BP changes, when Sagiv et al. (2006) studied adolescents exercising on a treadmill for 30 min (LC = 333 g kg^−1^ of body mass) SBP was elevated from 129.8 to 147.7 mmHg, diastolic blood pressure (DBP) remained unchanged. In another adolescent study, SBP continued to significantly increase with increasing loads, while DBP remained the same until LC of 15% body mass was used (Hong et al., 2000). These changes in BP in regards to LC and exercise have been found by other researchers as LC tends to have a lesser effect on DBP but significantly increases SBP (Miller et al., 2002; Ribeiro et al., 2014; Zhao et al., 2014). While further research is needed to determine a more precise LC mass, placement of this mass, speed, and gradient to avoid high cardiovascular strain, it is clear all these may synergistically increase the demands of the CVS and hinder exercise/military performance.

## 6 RESPIRATORY EFFECTS OF LOAD CARRIAGE

In addition to the deleterious CVS effects LC places upon the individual, the mere placement of LCs personal protective equipment (ruck, body armor) upon the chest wall (thoracic cavity) impedes another critical physiological system's function and capacities; i.e. the respiratory system (Shei et al., 2017). Under normal conditions (no chest wall restriction), the respiratory system utilizes pressure and flow gradients during a standard respiratory cycle (inspiration and expiration) to match the metabolic demands, both at rest and during physical activity, to facilitate the delivery of oxygen to tissue. Early work from the 1950 s outlined this respiratory system phenomenon as a balance between the mechanical work performed by the respiratory muscles to overcome elastic forces of the thoracic cavity (Otis et al., 1950) and the total energy cost per breath, which derives a value of work of breathing (*W*_b_, the mechanical work per breath [Joules]) and power of breathing (*P*_b_, the power generated by the respiratory muscles [Joules min^−1^]) (Cross et al., 2021; Otis, 1954).

Nevertheless, foundational work nearly three decades later expanded beyond the actual physiological *W*_b_ phenomenon by revealing that loading the chest wall (CWR), to the point where forced vital capacity was reduced by 40%, created greater inspiratory muscle workloads with resultant increases in breathing frequency (*f*_b_) and inspiratory flow rates, which ultimately leads to diaphragmatic fatigue (RMF) during high exercise intensities (Tomczak et al., 2011). Interestingly, the same result was not displayed with abdominal cavity restrictions alone where forced vital capacity (FVC) was reduced by 13% (Hussain & Pardy, 1985). These disparities could be explained by methodological differences between the studies including variations in the level of restriction imposed by the CWR device. Another consideration is that abdominal pressures increase during expiration (due to increasing abdominal muscle recruitment), which alters breathing mechanics and decreases diaphragmatic contractility, with a subsequent reduction of exercise time during high‐exercise intensities (Hussain & Pardy, 1985).

Coast et al. (1990) showed through inspiratory pressure measures that the greater the aerobic capacity of the individual (fitness level) the greater one's ability to stave off RMF during maximal exercise; thus, the respiratory muscles display a similar characteristic of adaptability like other skeletal muscle tissue. However, a consequence of CWR is that it increases the energy cost of inspiration and limits maximal exercise capacities and performance outcomes (Coast & Cline, 2004; Gonzalez et al., 1999). It is noted that the reductions in performance outcomes reported by Coast and Cline (2004) were observed when the CWR device reduced FVC by 7% to 12%, which is similar to the reductions in FVC observed with loads ranging from 10 to 47 kg (Armstrong et al., 2019; Armstrong & Gay, 2016; Bygrave et al., 2004; Dominelli et al., 2012; Majumdar et al., 1997).

Brown and McConnell (2012) acknowledged that thoracic LC induces a volume limitation upon the thoracic cavity which impedes operational lung volumes and hinders the operating function of the respiratory system. Consequently, the increased demand placed on the inspiratory muscles contributes to the premature development of RMF. Moreover, lung volume constraints resulting from LC forces the respiratory muscles to work on a proportion of their length‐tension curve that is inefficient (Brown & McConnell, 2012). In terms of the specific effects of how restrictive loads may impact pulmonary function, both Armstrong et al. (2019) and Dominelli et al. (2015) observed altered operational lung volumes and function of the respiratory system as breathing mechanics become impaired with LC. Even at rest, pulmonary function declines with incremental loading with a backpack (0–50 kg), as Dominelli and Sheel (2012) observed an 8% reduction in forced vital capacity (FVC) when wearing a 35 kg backpack, while Armstrong and Gay (2016) found 4%–6% reductions in FVC and FEV_1_ in body armours weighing 8–10 kg. Armstrong et al. (2019) found that the reduction in FVC and FEV_1_ increased as torso bore load mass was increased from 12 to 47 kg; the authors reported reductions ranging from 8% to 15% and 6% to 14% for FVC and FEV_1_ respectively. Reductions in vital capacity (VC) are not observed but, during periods of high‐intensity work, the ventilatory demand is increased 10 to 15‐fold which has detrimental effects on the ventilatory reserve. During normal respiration, tidal volume (*V*_T_) increases initially at lower workloads by a decrease in end‐expiratory lung volume, which serves to optimize diaphragm length (Aliverti, 2008). This understanding has been furthered during dynamic exercise where the use of thoracic restriction and occupationally relevant equipment (breathing apparatus) have been used. Self‐contained breathing apparatus (SCBA) systems are commonplace in occupational groups (firefighters) and are used to protect the respiratory system during occupational tasks, but also have deleterious results as they impede maximal ventilation (Butcher et al., 2007). These systems, typically worn upon the thorax, use a greater proportion of end‐inspiratory lung volume (EILV) and increase the elastic *W*_b_ (~59%) at rest and during sub‐maximal exercise at 240 W; no difference was observed at exercise intensities <240 W. The exact mechanism is not known but it has been proposed that the presence of a positive pressure surrounding the face mask adds a resistive load to breathing (Butcher et al., 2007). Although under certain environmental conditions, assistive positive pressure is not as much of a hindrance to expiratory flow and can benefit the inspiratory function via an unloading effect (explained eloquently by Dominelli et al., 2016), in other cases, this may be detrimental. For instance, increased *W*_b_ causes fatigue of the respiratory muscles, exacerbates the dyspnoea response to exercise with SCBA and limits exercise performance during short duration (~5 min) exercise tasks (Butcher et al., 2007).

Restriction of the chest wall, without the addition of external loading of mass, has also been used inelastic strapping (Miller et al., 2002) or fiberglass air‐pressurized chest‐casting (Cline et al., 1999) to assess cardiorespiratory function. At rest and during submaximal cycling, Miller et al. (2002) found that at 25% and 45% of peak power output, total lung capacity was decreased by 33%, VC was decreased by 38%, residual volume was decreased by 23% and both peak expiratory and peak inspiratory flow rates were reduced. Interestingly this also resulted in reduced *V*_T_ and increased f_b_ to coincide with increased work done by the diaphragm, which saw the biggest rise in gastric pressure. Subsequently, cardiac output was reduced during exercise (~10%–12%) primarily due to reduced stroke volume (~16%) while HR was unchanged (Miller et al., 2002). Similarly, inelastic strapping demonstrated specific fatigue of the diaphragm following sub‐maximal cycling exercise at 45% peak power output (Tomczak et al., 2011). Inelastic straps were used to restrict FVC to 40%; diaphragm contractions were measured using cervical magnetic stimulation of the phrenic nerve coupled with the measurement of transdiaphragmatic pressure. Both potentiated and non‐potentiated twitches were used to assess fatigue of the diaphragm at 10‐ and 30‐min post‐exercise. During exercise with inelastic strapping, the *W*_b_, respiratory pressures, ventilatory parameters and perceptions of respiratory and leg discomfort were increased compared with the control trial along with concomitant reductions in diaphragm contractility during non‐potentiated (20.2% ± 15.3%) and potentiated twitches (23.3 ± 12.4%). The magnitude of diaphragm fatigue observed was correlated with the inspiratory elastic *W*_b_ (*r* ^2^ = 0.74) and it was concluded that fatigue of the diaphragm occurs under restricted conditions during submaximal exercise (Tomczak et al., 2011). These studies highlight the implications and the extent to which thoracic restriction and altered breathing mechanics can have on breathing parameters, however, the validity of this work does not translate directly toward occupational tasks given that the levels of restriction induced by the CWR method are representative of disease states rather than LC.

More recent work has consistently demonstrated that exercise with LC reduces the force‐generating capacity of the respiratory muscles (Armstrong et al., 2019; Faghy & Brown, 2014a, 2019). Faghy and Brown (2014b) reported that *P*_Imax_ was reduced following 60 min of LC at 6.5 km h^−1^ relative to resting values (range 11%–13%) and again following a 2.4 km self‐paced time trial relative to post LC values (range 3%–5%) and resting values (range 13%–17%). In comparison, there were no reductions in *P*_Ima*x*_ during an identical trial without a load (post 60 min 1%, post 2.4 km time trial 4%). Similarly, Shei et al. (2018) demonstrated a reduction in non‐potentiated diaphragmatic twitch pressures following a running bout to volitional exhaustion with a 10 kg load. In an unloaded control run to exhaustion at matched *V̇*O_2_, (70% *V̇*O_2max_), diaphragmatic fatigue was not observed. The exact mechanisms underpinning these findings are yet unknown, however, both elevated *W*_b_ and impaired breathing mechanics imposed by both chest wall loading and restriction (Dominelli et al., 2016; Tomczak et al., 2011) likely play an important role. Moreover, an increase in dead space ventilation, observed by both Phillips, Stickland, & Petersen (2016) and Shei et al. (2018), may also contribute to more severe RMF with LC.

In addition to forcing the respiratory muscles to work in a sub‐optimal portion of their length‐tension curve and potentially elevating *W*_b_, reductions in operating lung volumes imposed by LC may also cause tidal airflow rates during the respiratory cycle to reach the maximal limit imposed by mechanical constraints such as airway size and intrathoracic pressures, potentially resulting in expiratory flow limitation (EFL). Although Shei et al. (2018) did not observe greater EFL with a 10 kg load during sub‐maximal running at 70% of *V̇*O_2max_, it is possible that at greater ventilatory workloads, EFL may be exacerbated by differing LC characteristics. Mechanical constraints to ventilation may contribute to reduced ventilatory reserve with LC, and consequent impairment to exercise capacity.

It is well established that exercise with LC increases the *W*_b_ through a curvilinear increase in the force and velocity of contraction (Brown & McConnell, 2012). This increases the work done by the inspiratory musculature for a given breath, which occurs through changes in EILV, end‐expiratory lung volume, and the addition of the restrictive component of the load, imposed by the shoulder straps, which also reduces operational lung volumes. In combination, further reductions in the efficiency of the respiratory musculature occur as their position on their length‐tension curve becomes suboptimal and subsequently leads to reduced contractile efficiency (Dominelli et al., 2012). The nature and placement of external loads upon the thorax increases work done (often demonstrated via increased total energy expenditure), whilst chest wall restriction and impaired respiratory muscle and lung function remain plausible explanations, global changes in physiological function may be augmented by changes in metabolic and cardiovascular exercise profile are also important. This was highlighted in a previous study where chest wall loading reduced lung function variables (FEV_1_, FVC and FEV_1_/FVC) however, no within exercise differences in cardiopulmonary, perceptual responses or performance were observed (Giuriato et al., 2020). In the context of load carriage exercise tasks, it may therefore be difficult to isolate the direct physiological effects of load carriage tasks for a single system (e.g., impaired chest wall mechanics/respiratory muscle performance) where increases in physiological strain is part of a global physiological responses to a given exercise task (e.g., metabolic/cardiopulmonary consequences of increased physical task demands).

## 7 EFFECTS OF LOAD CARRIAGE ON EXERCISE CAPACITY

Prolonged engagement in LC activities in occupational settings can last for several hours and involve repeated bouts of exercise. Recognizing a lack of ecological validity in previously developed protocols, Faghy and Brown (2014b) devised a laboratory protocol to determine the effects of LC exercise on exercise capacity. This protocol was based on the UK Annual fitness test for Infantry soldiers (Treweek et al., 2019) and incorporated repeated bouts of exercise. Sixty minutes of carrying 25 kg in a backpack during sub‐maximal walking exercise (6.5 km h^−1^) demonstrated an exacerbated physiological response and notably caused significant fatigue of the respiratory muscles. RMF was further increased following the completion of a 2.4 km best‐effort march. Additional investigations demonstrated that sub‐maximal walking with no load or carrying 10, 15 and 20 kg in a backpack did not cause RMF despite an increase in physiological, metabolic and perceptual parameters (Faghy, 2016; Faghy et al., 2016). These findings were likely influenced by the configuration of the load carried as RMF was subsequently observed following 50 min of marching (4.9 km h^−1^) with 11 kg body armor (Armstrong et al., 2017).

Shei et al. (2018) observed a 42.9% reduction in constant work rate (70% *V̇*O_2peak_, absolute *V̇*O_2_ matched for LC and unloaded control) time to exhaustion with LC compared with an unloaded control. Changes in constant load exercise tasks could be attributed to reduced neuromuscular function (Blacker et al., 2013). Reductions in the force‐producing capability of a muscle have negative neuromuscular and metabolic consequences (Byrne et al., 2004), therefore, if the neuromuscular function is compromised in the days following LC, an individual's physical performance is likely to be impaired and the risk of injury increases (Hughes et al., 2019; Knapik, 2014; Schuh‐Renner et al., 2017). This has relevance in occupational settings as, following LC, participants are often required to undertake additional physically demanding and skilled tasks such as setting up and use of equipment, marksmanship (Hadid et al., 2017) military‐specific tasks (Knapik et al., 2004) or additional bouts of LC (Blacker et al., 2013).

During human locomotion and LC activities, the basic muscle function is the stretch‐shortening cycle, where the pre‐activated muscle is first stretched (eccentric action) and then followed by the shortening (concentric) action. Neuromuscular impairment is greatest following eccentric contractions, the pattern of which has been suggested to be bimodal (Dousset et al., 2007). Typically, there is an immediate reduction in the force production of a muscle, with a small recovery within 1–2 h that is followed by a secondary reduction, where recovery can last for between 4 and 8 days depending on the severity of the exercise bout (Dousset et al., 2007). Blacker et al. (2013) investigated changes in neuromuscular function following 120 min of LC during level treadmill walking whilst also investigating the changes using voluntary and electrically stimulated contractions 0, 24, 48 and 72 h after LC. During an unloaded control trial, no changes in neuromuscular function were observed after walking for 120 min at 6.5 km h^−1^ on a 0% gradient. However, the addition of carrying a 25‐kg backpack caused reductions in the levels of force produced by the knee extensors during both isokinetic and isometric contractions; findings were still apparent up to 72 h following the LC bouts. Similarly, O'Leary et al. (2018) investigated between‐sex differences in British Army recruits in response to 9.7 km loaded marching (15 or 20 kg) and found that marching reduced knee extensor isometric MVC force and vertical jump height in both sexes. Sex differences in respect of LC exercise and performance will be explored explicitly below.

Previous work in this area has focused on the use of submaximal protocols; however, operational tasks also involve maximal exercise. Literature examining the effects of maximal exercise trials to date is limited.

## 8 SEX‐BASED DIFFERENCES IN WORK OF BREATHING AND LOAD CARRIAGE PERFORMANCE

In considering exercise performance with LC, sex‐based differences between women and men are an important consideration, especially the aspect of *W*_b_ and the consequences of high‐intensity endurance exercise. Presently, it is understood that regarding sex‐based differences in *W*_b_: (1) women have a greater total *W*_b_ at absolute ventilation and for a given workload to body mass ratio due to greater resistive *W*_b_ caused by smaller airways in women when compared to men plus breathing patterns favoring increased ƒ_b_ (Guenette & Sheel, 2007; Sheel & Guenette, 2008); (2) per the pressure–time product of the diaphragm/pressure–time product of the esophagus, the diaphragm in women might be more resistant to RMF relative to men during endurance exercise (Guenette et al., 2010; Hunter, 2016); (3) for submaximal and maximal exercise intensities, respiratory muscle oxygen uptake ($\dot{V}$O_2RM_) is significantly greater in women compared with men (Sheel & Guenette, 2008); and (4) during heavy intensity exercise the $\dot{V}$O_2RM_ represents a greater fraction of whole‐body oxygen uptake in women that may have repercussions for the integrated physiological response (Dominelli et al., 2015; Sheel et al., 2016).

Orr et al. (2011) previously summarized specific considerations for women who are required to carry a load. Factors such as biomechanical and physiological impact, implications to health and the “female triad.” Interventions such as equipment fitting, nutrition, and strength and conditioning programs were highlighted as important considerations to mitigate sex‐based differences in LC performance (Orr et al., 2011). As previously discussed, LC induces a restrictive thoracic ventilatory limitation (CWR) that increases the elastic *W*_b_, rendering the respiratory muscles vulnerable to fatigue with a concomitant reduction in exercise tolerance, which may impose critical limitations on certain occupational duties (LC tasks) (Brown & McConnell, 2012). Despite recent advancements, there still exists a lack of understanding of sex‐based differences in RMF limitations on human performance and the potential negative impact on neuromuscular function/fatigability with the prevailing mechanisms and the functional consequences (Hunter, 2016). Recent research has provided new results on the potential impact of respiratory system function, ventilatory responses, and muscle fatigue during LC performance (Faghy & Brown, 2014b; Phillips, Stickland, & Petersen, 2016; Shei et al., 2018); however, a limitation of these studies was the exclusion of women and the limited assessment of more robust respiratory measurements such as *W*_b_. Hunter (Hunter, 2016) subsequently postulated that dissimilar skeletal muscle contractile mechanisms between women and men could explain variances in muscle fatiguability and that these differences are task‐specific. Thus, it is too simplistic to assume that people should be treated comparably—especially relative to LC tasks. In the UK army, the exclusion of women in ground close combat (GCC) roles was lifted in 2016. From an applied science and operational perspective, some important information has been discovered characterizing the impact of sex‐based differences; however, an integrative approach toward the systematic study across research questions to discover the mechanisms of human performance limitation, such as physiological system(s) function, anthropometrics, biomechanical efficiency; is needed to address the sex‐based differences knowledge gap that has not been identified clearly (Epstein et al., 2015; Greeves, 2015; Nindl & Sharp, 2015; Orr et al., 2014).

Compared to men, women have lower absolute respiratory muscle strength (Black & Hyatt, 1969; Gonzales & Scheuermann, 2006; Ozkaplan et al., 2005), smaller lung volumes, smaller diameter airways (Guenette & Sheel, 2007), lower oxygen‐carrying capacity, lower upper body strength and lower fat‐free mass (Åstrand et al., 2003; Knapik et al., 2004). Consequently, women typically have a poorer strength‐to‐body mass ratio (Orr et al., 2011) meaning that when LC mass is based upon an absolute load, the relative load carried is greater for women leading to a larger relative burden and increased relative intensity (Bhambhani & Maikala, 2000; Holewijn et al., 1992; Orr et al., 2011; Phillips et al., 2019). Additionally, sprint times in women are affected to a larger extent than in men (36% vs. 29%, respectively) as a result of the increased load (Treloar & Billing, 2011). This may reduce LC task duration and result in greater levels of discomfort for women, particularly if LC apparel/systems are based on the anthropometry of men (Knapik et al., 2004; Martin & Nelson, 1985).

The degree to which sex differences in LC performance reflect biological differences per se rather than anthropometrical differences is an important question and has implications for the performance of LC tasks. Because men tend to be heavier than women, absolute *V̇*_E_ (Bhambhani & Maikala, 2000; Phillips et al., 2019) and *V̇*O_2_ are greater in men when carrying upper body loads (≤40% of body mass or absolute loads of 12–50 kg) during walking (3–6.5 km h^−1^) exercise. However, when *V̇*_E_ (Vickery‐Howe et al., 2020) and *V̇*O_2_ (Bhambhani & Maikala, 2000; Godhe et al., 2020; Phillips et al., 2019; Vickery‐Howe et al., 2020) are expressed relative to body mass, no differences are evident. Nevertheless, women still work at a greater percentage of their *V̇*O_2max_ whether walking with identical relative loads based on body mass (≤40%) or walking with fixed‐mass loads (8–88 kg), regardless of whether the pace is fixed or self‐selected (Bhambhani & Maikala, 2000; Holewijn et al., 1992; Rice et al., 1996; Vickery‐Howe et al., 2020). Interestingly, this greater physical burden of LC exercise does not automatically result in a larger magnitude of muscle fatigue in women, even though *V̇*O_2_ may exceed the ventilatory threshold during lighter fixed‐load exercise (Bhambhani & Maikala, 2000). As stated earlier, O'Leary et al. (2018) found that the degree of knee extensor fatigue (assessed using isometric maximal voluntary contractions) was significantly less in women, and attributed this to a greater proportion of more fatigue‐resistant type I muscle fibers.

Heart rate and RPE (Bhambhani & Maikala, 2000; Godhe et al., 2020; Holewijn et al., 1992; Monod & Zerbib, 1985; O'Leary et al., 2018; Rice et al., 1996) are also increased in women when either absolute load or metabolic demand is fixed. However, when HR is expressed as a percentage of body mass, differences are reduced, disappearing altogether when expressed in relation to lean body mass (Monod & Zerbib, 1985). Likewise, RPE and dyspnea are similar between men and women when expressed at the same relative (percentage of *V̇*O_2max_) metabolic demand but are greater in women at comparable sub‐maximal (but an increased relative proportion of *V̇*O_2max_) metabolic demands (Phillips et al., 2019). Even when women are height‐matched to men, the former still exhibit a more rapid (increased *f*_b_) and shallow (lower *V*_T_) breathing pattern during LC tasks (Phillips et al., 2019). During peak exercise with load, it has been found that women encounter an increase in relative dead space and impaired alveolar ventilation compared to unloaded marching (Phillips et al., 2019). According to Phillips et al. (2019), the greater *W*_b_ experienced by women may prevent the respiratory muscles from compensating for carbon dioxide retention during peak, but not sub‐maximal, LC exercise.

Collectively, such observations suggest that many of the observable differences between men and women performing LC tasks can be reduced, and in some cases eliminated, by accounting for anthropometric and strength differences. This suggests that sex per se is not the dominant factor in explaining differences in LC performance between men and women. Instead, differences in anthropometry may play a larger role than inherent biological differences.

## 9 STRATEGIES TO AMELIORATE NEGATIVE EFFECTS OF LOAD CARRIAGE

**FIGURE 1:**
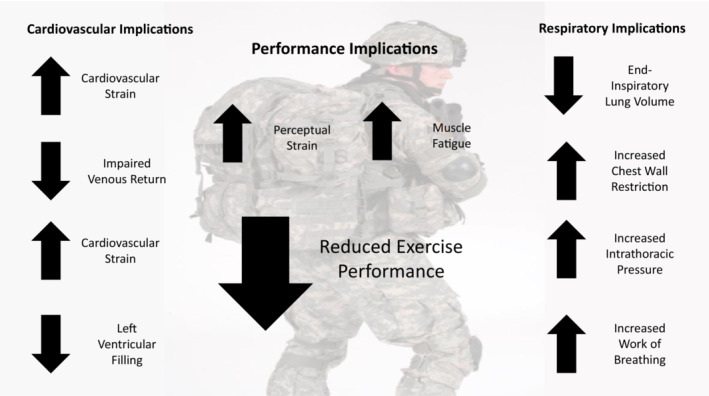
A summary of the cardiovascular and respiratory limitations imposed by load carriage activities and the impact this has on performance.

A summary of the cardio‐respiratory implication of load carriage performance are summarized in Figure 1. Physical training strategies have long been used to considerably improve the ability to perform physically demanding occupational activities and consist predominantly of muscular strength and endurance, and aerobic endurance (Knapik et al., 2012). Within military groups, basic training is designed to increase the necessary skills and the physical fitness standards ready for operational duty (Santtila et al., 2010). Literature in this area has covered extensively the requirements of programs to enable physical development but this has largely consisted of standardized requirements that are suited to large muscle mass activities and training of large groups (Harman et al., 2000). It is apparent from this review that greater thought and consideration are required in the development of physical training strategies to ensure that the physiological, biomechanical, and current importance of psychological components of training is covered adequately.

LC training protocols that combine aerobic training with strength training (upper and lower body) have proved efficacious. However, it is noted that none of these protocols involves targeted training of the respiratory muscles using inspiratory muscle training (IMT). Given the impact of LC on ventilatory function, the authors believe that training the respiratory muscles will also prove beneficial to LC performance (Shei, 2018). Within the literature, the use of IMT methods has been devised and investigated in relation to improving respiratory muscle strength and as a consequence whole body performance during exercise with LC (Faghy & Brown, 2016, 2019; Shei et al., 2018).

IMT methods possess similar training principles to those of other more superficial skeletal muscles that are readily accessible and respond to regular training via structural adaptations (McConnell, 2009). The use of these protocols has demonstrated the ergogenic benefit to LC performance throughout either 4 or 6‐week interventions (Faghy & Brown, 2016, 2019). Following 6 weeks of pressure threshold loading (2 × 30 breaths at 50% *P*_Imax_) 2.4 km time‐trial performance was improved by 8 ± 4% (absolute reduction = 1.3 ± 0.7 min). Similarly, Shei et al. (2018) observed a 29.3% improvement in constant work rate (70% *V̇*O_2max_) time to exhaustion with LC following 6 weeks of flow resistive IMT. Improved performance on LC tasks is primarily the result of increased inspiratory muscle strength which permits the inspiratory muscles to work at a lower relative intensity during exercise tasks (Turner et al., 2012). To date, research has demonstrated a multifaceted effect of IMT on physiological systems and whole‐body performance which includes a reduction in HR, during fixed‐intensity submaximal exercise (Faghy & Brown, 2016, 2019). Reduced HR occurs because of diaphragmatic contractions occurring at reduced relative intensities, thus reducing cardiovascular demand during exercise. Second, to this, normalized intra‐thoracic pressure swings during the breathing cycle, reduce the work of the diaphragm during exercise, thus the respiratory muscles command reduced levels of cardiac output (Miller et al., 2002). Third, reduced perceptual responses have been observed following LC. The likely mechanism of attenuated RMF is a reduction in discharge frequency of mechano‐sensitive type III and IV nerve afferents, which project to the sensory cortex (Dempsey et al., 2006). This is caused by reduced metabolite accumulation that stimulates the chemically sensitive afferents and reduces afferent feedback and effort perceptions during exercise tasks (Sinoway et al., 1993).

Despite the ergogenic effect of IMT in relation to exercise performance, *P*_Imax_ and other physiological responses, IMT interventions to date have failed to attenuate muscle fatigue; therefore, the true ergogenic effect of IMT may have not been realized. The attenuation of RMF is a direct contrast to previous IMT studies that show attenuated inspiratory muscle fatigue after cycling time trial exercise (Romer et al., 2002). One consideration here is the additional non‐respiratory roles of the breathing muscles that are tasked with supporting posture and spinal stability (Roberts et al., 2018). Thoracic LC increases postural sway and the reliance upon the diaphragm to stabilize the spine (Hodges et al., 1997). In addition, LC fatigues accessory respiratory muscles of the thorax which support pulmonary ventilation during exercise with high breathing demands (Blacker et al., 2010). Therefore, it is possible that IMT failed to attenuate fatigue that is associated with the non‐respiratory functions of the inspiratory muscles. It is imperative to consider the respiratory muscle contribution to both ventilation and non‐respiratory roles (movement and maintaining posture). For example, the addition of external loads on the thorax will place extra stress on the abdominals to maintain posture and will challenge spinal stability as load mass increases. Accordingly, it is suggested that traditional IMT protocols adopted to date, i.e., where controlled and repeated inspiratory efforts are performed when not under thoracic restriction, may not target sufficiently the length‐tension characteristics of the primary and synergistic respiratory musculature adopted during LC exercise (Brown & McConnell, 2012) and is something that should be considered in future work.

## 10 LOAD CARRIAGE FAMILIARITY

Another key facet to consider when thinking of strategies to mitigate the negative consequences of LC activities is the “experience level” of the individual that will be performing LC. Operationally, an Australian Government, Department of Defense technical report (Drain et al., 2012) represents this point, categorically, within an individual's own “LC capacity” and lists “LC experience” as an important factor, which commanders should take into consideration while mission planning. From a research perspective, the distinction of experience has knowingly, and with meaningful intent, been part of selection criteria and comparison strategies within findings, to minimize the risk of misinterpretation and inappropriate application. For instance, Wang et al. (2012) made notable mention of their selection of a college student with “…no experience of military basic training…” and “…no experience of walking under the influence of fatigue…”, therefore making it difficult to draw definitive conclusions relating to familiarity and training experience. Additionally, Lidstone et al. (2017) explicitly state that the results of their “inexperienced population” is limited in generalizability and should not be indicative of what could be seen in experienced US soldiers, with the need for future research to evaluate the physiological and biomechanical responses to LC between experienced active‐duty personnel and inexperienced military recruits. An important assessment of LC biomechanics by Seay (2015) reported that recent insights into LC kinematic responses were experienced‐dependent with cadence‐related versus load‐related adaptations occurring between inexperienced trainees versus experienced soldiers, respectively.

Future LC studies which target occupational groups must consider the end user during study design. The study participants should be representative of the target population in terms of their LC familiarity to ensure that the research can be translated directly to the end users. Further, interventions designed to mitigate the detrimental effects of LC should be tested in the target population before implementation. This will validate the benefits of the intervention and facilitate end‐user feedback to ensure that the intervention can be implemented successfully.

## 11 FUTURE RESEARCH DIRECTIONS

Although a significant amount of research has established our current LC knowledge base, this review article has also revealed considerable research gaps within the literature and highlighted important future scopes of work. Poignant to the effects of carrying thoracic loads upon the respiratory system and subsequent performance, a real understanding of the impact of cumulative CWR on *W*_b_ during LC and resultant biomechanical compensatory alterations has not been investigated. There is a need for more interdisciplinary research that is focused on optimizing the preparation and performance of a single population. What we do know is the *W*_b_ and RMF are major determinants of endurance exercise performance through critical governance via the metaboreflex control mechanism of the locomotor muscles. The relationship between personal and protective equipment is established and induces CWR and subsequent RMF. However, whether RMF during LC performance is sufficient to trigger the metaboreflex is yet to be determined. Further investigation might seek to determine the impact of progressive scaling‐mass of combat equipment under various configurations (various body armor fittings or plate configurations, different loads carried for sustainment versus assault operations) on CWR, the *W*_b_ and RMF and how this might modify the potentially dire consequences on LC performance. Future attention should also be directed to the use of targeted LC training that incorporates IMT methods and, given the potential postural and stability role of the expiratory muscles, RMT. Particular attention should also be paid to equipment modification and development to reduce the demand for the respiratory musculature and to assess the benefits of more equal distribution and the impacts on LC performance.

## 12 CONCLUSIONS

Research demonstrates the considerable physiological challenge associated with LC activities and highlights the use of successful interventions to improve LC performance. Whilst, considerable attention has been paid to this area of research, continued approaches are needed to further increase the physiological understanding and implications of LC activities concerning the work of breathing to inform the design and development of load carriage equipment and efficacious intervention strategies that enhance performance on LC tasks. Future studies should be designed with a focus on the population of interest; high‐quality research on women is identified as a significant data gap that needs to be addressed to ensure that research in this field reflects the diversity of occupational groups.

## AUTHOR CONTRIBUTIONS

Mark A. Faghy: Conceived the idea for the manuscript with Ren‐Jay Shei and Mitch Lomax and led the writing of several sections of the manuscript and integrated author contributions in preparation for submission. Ren‐Jay Shei: Conceived the idea for the manuscript with Mark A. Faghy and Mitch Lomax and contributed to numerous areas of the writing project. Ren‐Jay Shei reviewed and approved the manuscript prior to submission. Nicola C. D. Armstrong: Nicola C. D. Armstrong contributed to the writing and revisions of the submission and approved the submission. Mark White: Mark White contributed to the writing and revisions of the submission and approved the submission. Mitch Lomax: Conceived the idea for the manuscript with Mark A. Faghy and Ren‐Jay Shei and contributed to areas of the writing project. Ren‐Jay Shei reviewed and approved the manuscript prior to submission.

## ETHICS STATEMENT

Not required as part of a review.
